# Preface to Special Topic: Ultrafast Structural Dynamics—A Tribute to Ahmed H. Zewail

**DOI:** 10.1063/1.4998273

**Published:** 2017-08-10

**Authors:** Majed Chergui

**Affiliations:** Ecole Polytechnique Fédérale de Lausanne, Laboratoire de Spectroscopie Ultrarapide and Lausanne Centre for Ultrafast Science (LACUS), ISIC, Faculté des Sciences de Base, Station 6, CH-1015 Lausanne, Switzerland

Ahmed Zewail passed away on 2 August 2016. This was a tremendous loss to the community of scientists working on ultrafast phenomena. He pioneered the study of the dynamics of chemical bonds using ultrashort laser pulses, which he had named Femtochemistry. This achievement would change our way of looking at how chemical bonds break, form, and transform. It would encompass several fields of research beyond chemistry, basically all areas of science where the chemical bond is present, i.e., materials science and biology.

Femtochemistry allowed atomic motion to be observed in “real-time,” i.e., at the atomic-scale of time, the femtosecond. This also rose the awareness of the importance to visualize the dynamics of assemblies of atoms at the atomic-scale of space, i.e., the Å. For this, one needed short wavelength radiation, i.e., either electrons or X-rays. Ahmed Zewail started, in the early 1990s, a second revolution by taking up the colossal task of implementing ultrafast electron scattering and diffraction and then later, ultrafast electron microscopy and spectroscopy. Within 10–15 years, he brought the methodology to the point that he could investigate a wide variety of systems and phenomena and generated an emulation in the community, comparable to his popularization of Femtochemistry in the 1990s.

This special Issue of the journal *Structural Dynamics* was decided within a few days of his passing and I was moved by the immediate and overwhelming response of friends and colleagues of Ahmed who immediately accepted to contribute and did so. This is a tribute to his great contributions to Science. It is also a testimony of the profound influence he has had on the broader scientific community and of the diversity of his interests and achievements.

The issue contains a selection of articles by world leading experts in ultrafast optical, electron, and X-ray based methods, and in materials science, in the photophysical and photochemical dynamics of molecular and biological systems and in liquid phase dynamics. It opens with a biographical review of his works that led to the birth of Femtochemistry and on the influence the latter had on the birth of Ultrafast Structural dynamics, focussing on his major achievements in the implementation and use of ultrafast electron techniques. It is followed by two Perspective articles, whose contents—the description of solvation shell of proteins and its dynamics, and the probing of 2D Biosystems with ultrashort X-ray pulses—reflect well Ahmed Zewail's breadth of interests.

A large number of articles in this issue deal with electron-based methods and many of these are by former members of Zewail's team. As a consequence, it is also no wonder that several articles deal with materials, since a good part of Ahmed Zewail's activity in the past 12–15 years had shifted towards this field. Finally, many articles deal with materials and with molecular or biological systems investigated with a diversity of tools ranging from the X-ray domain through the vacuum ultraviolet to the infrared domain. The diversity of methods and scientific results presented in this issue is a testimony of the deep mark left by Ahmed Zewail in Science as his contributions have ramifications in very diverse fields of research.

Ahmed Zewail was a member of the Advisory Editorial Board of Structural Dynamics. He had enthusiastically accepted my invitation to join the Board in 2014, even though he was already ill and had to reduce his activities. Despite this, he published literally an article every year in the journal. This Special Issue is dedicated to him. It is published with the generous support of the Lausanne Centre for Ultrafast Science (LACUS) at the Ecole Polytechnique Fédérale de Lausanne (EPFL), Switzerland. Ahmed Zewail was an Honorary Doctor of the EPFL. I also thank the Associate Editors of the journal for their help and assistance in processing the articles and the AIPP staff for their quick and efficient handling of the articles.

Nothing can better demonstrate that Ahmed Zewail's spirit is still alive among us than such tributes to his great achievements in Science and to his memory.

